# Supplementary material for: Comparative genomic analysis of thermophilic fungi reveals convergent evolutionary adaptations and gene losses
Source: Commun Biol. 2024 Sep 12;7:1124. doi: 10.1038/s42003-024-06681-w (PMC11393059; doi:10.1038/s42003-024-06681-w)
Supplement: Supplementary file 1 — Description of Additional Supplementary Materials [file 42003_2024_6681_MOESM1_ESM.pdf]

Description of Additional Supplementary Files

File name: Supplementary Data 1

Description: The 79 genomes used in this study and their respective metadata and references.

File name: Supplementary Data 2

Description: Counts of CAZymes families on the 79 strains used in the study

File name: Supplementary Data 3

Description: Orthologous clusters resolved by gene and species tree reconciliation and their respective annotation. Gene expansions and contractions based on CAFE. Machine learning training sets and prediction results.

File name: Supplementary Data 4

Description: Endoxylanases GH10 protein features.

File name: Supplementary Data 5

Description: MESQUITE complete results, phylogenetic species tree, and endoxylanase GH10 gene tree in newick format.
